# Supplementary material for: Model‐guided development of an evolutionarily stable yeast chassis
Source: Mol Syst Biol. 2021 Jul 22;17(7):e10253. doi: 10.15252/msb.202110253 (PMC8297383; doi:10.15252/msb.202110253)
Supplement: Supplementary file 1 — Appendix [file MSB-17-e10253-s002.pdf]

## Appendix

# Model-guided development of evolutionarily stable yeast chassis

Filipa Pereira[1,§], Helder Lopes[2,§], Paulo Maia[3], Britta Meyer[4], Justyna Nocon[1], Paula Jouhten[1], Dimitrios Konstantinidis[1], Eleni Kafkia[1,6], Miguel Rocha[2], Peter Kötter[4], Isabel Rocha[2,5*] and Kiran R. Patil[1,6*]

[1] Structural and Computational Biology Unit, European Molecular Biology Laboratory, Heidelberg, Germany

[2] Centre of Biological Engineering, Department of Biological Engineering, University of Minho, Braga, Portugal

[3] Silicolife - Computational Biology Solutions for the Life Sciences, Braga, Portugal

[4] Johann Wolfgang Goethe-Universität, Frankfurt am Main, Germany

[5] Instituto de Tecnologia Química e Biológica, Universidade Nova de Lisboa, Portugal

[6] The Medical Research Council Toxicology Unit, University of Cambridge, Cambridge, UK

* To whom correspondence should be addressed. Email: irocha@itqb.unl.pt; kp533@cam.ac.uk

**This PDF file includes:**

Appendix Figures S1 to S4

Appendix Tables S1 to S11

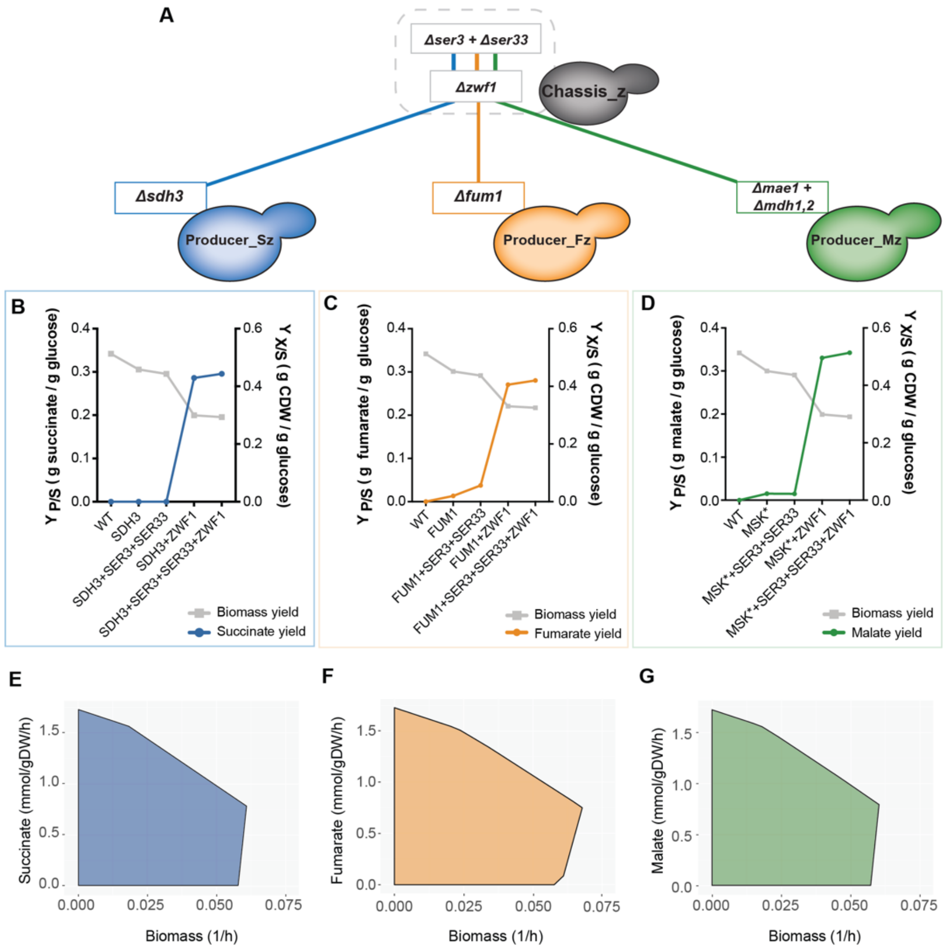


**Appendix Figure S1**. **Predicted effect of gene deletion targets for C4-dicarboxilic acids production.**

**A.** Illustrates the proposed strategy, simulated *in silico*, for chassis strain design (deletion of *ZWF1*, *SER3* and *SER33*) as well as the additional knockouts required for succinate (*SDH*-complex), fumarate (*FUM1*) and malate (*MDH1*, *MDH2* and *MDH3*) overproduction.

**B, C, D.** Panels Growth-coupling impact of the predicted knockouts on succinate (**B**), fumarate (**C**) and malate (**D**) production at product yield and biomass yield predicted using pFBA and iMM904. Only the combinations of knockouts selected for *in vivo* implementation are presented. WT stands for wild-type and MSK* stands for *MAE1*+*MDH1*+*MDH2* the "minimal set of knockouts" required for malate production.

**E** Predicted 'Production envelop' for succinate production in the *in vivo* implemented solution (*Δser3,33Δzwf1Δsdh3*).

**F** Predicted 'Production envelop' for fumarate production in the *in vivo* implemented solution (*Δser3,33Δzwf1Δfum1*).

**G** Predicted 'Production envelop' for malate production in the *in vivo* implemented solution (*Δser3,33Δzwf1Δmae1Δmdh1,2*) with impaired fumarate drain.

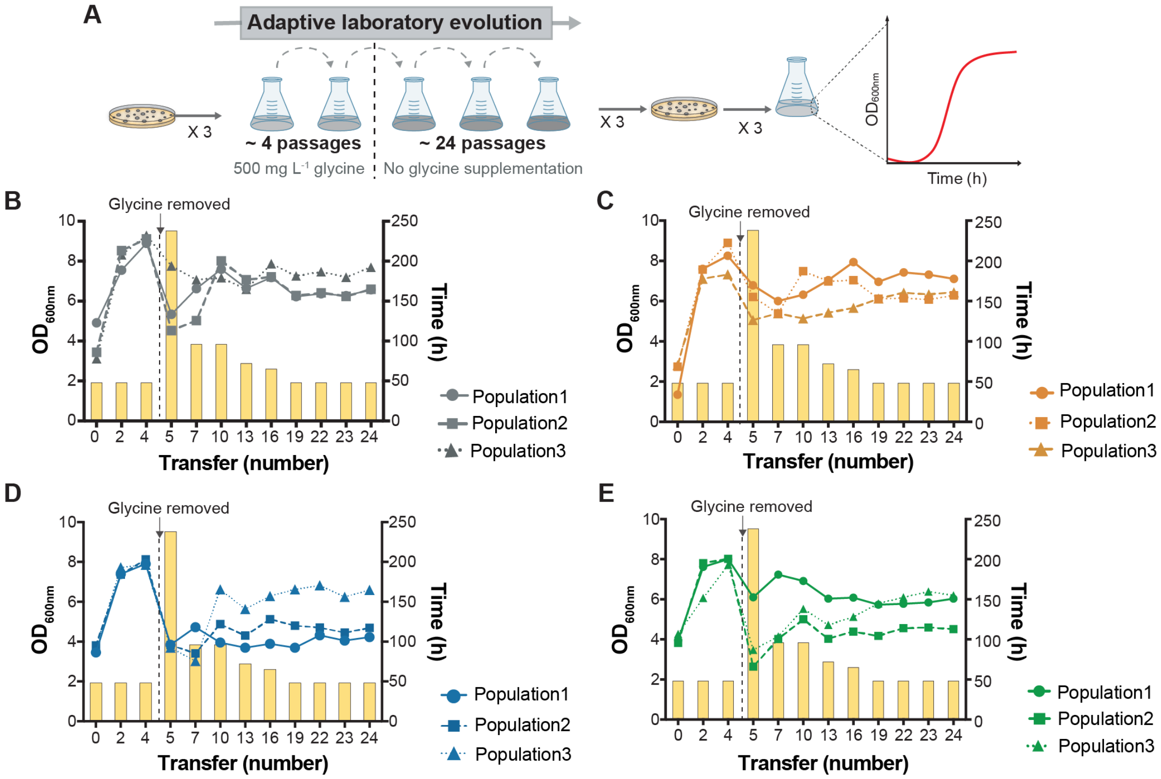


**Appendix Figure S2. Adaptive laboratory evolution experimental approach and fitness profile of the paralleled evolved populations during ALE.**

**A.** Scheme of the ALE experimental set-up and isolation of final evolved strains.

**B,C,D,E.** Fitness profile of paralleled evolved populations. For each population, The $OD_{600}$ and the cultured time (h) were recorded - for all transfers, before transferring it into fresh media. The three independent populations of all parental strains are represented by round, triangle and square symbols for Chassis_z (**B**), Producer_F (**C**), Producer_Sz (**D**) and Producer_Mz (**E**).

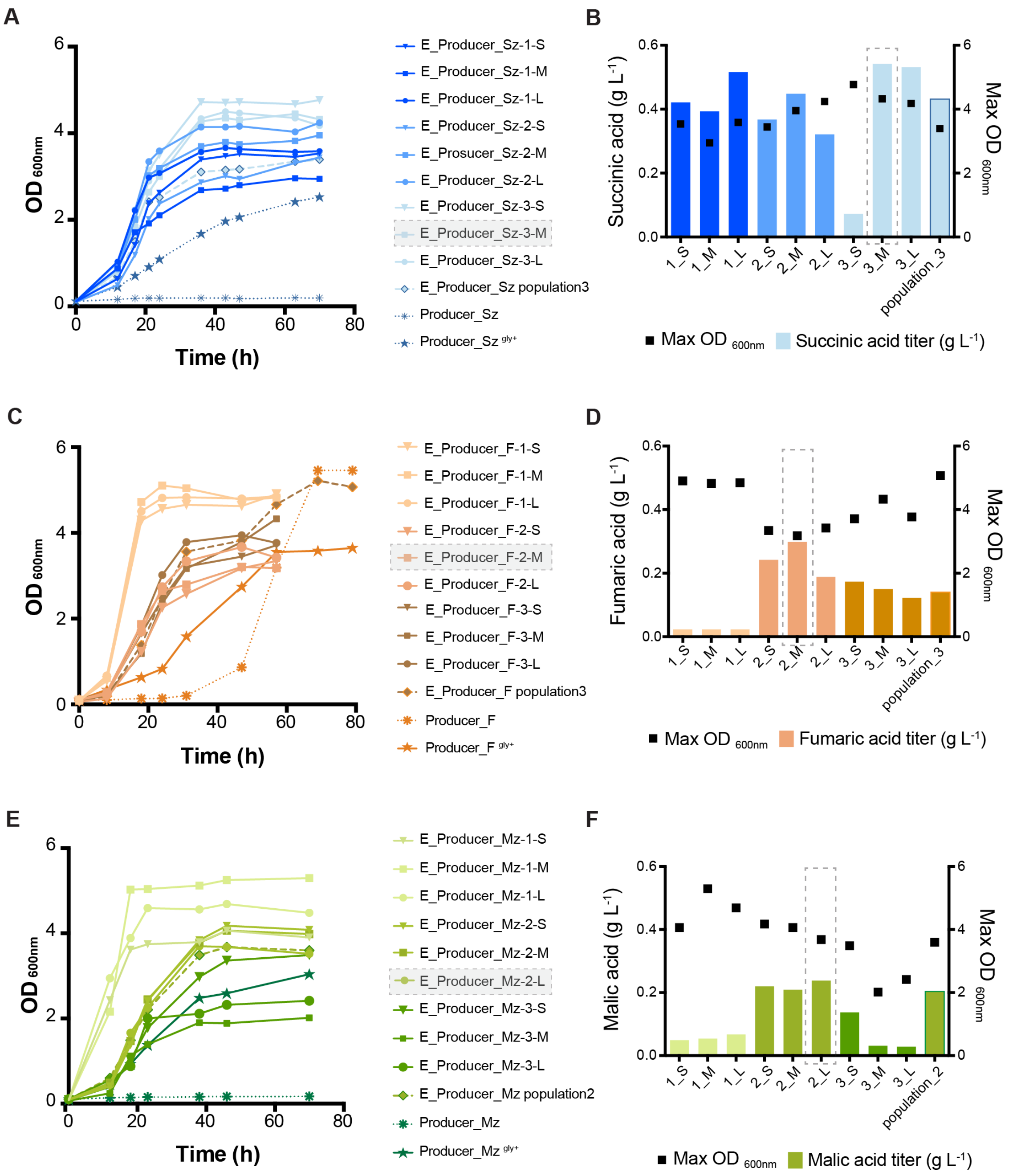


**Appendix Figure S3. Screening of three isolated strains from evolved populations.**

**A,B.** Growth profile (**A**) and succinate production for evolved Producer_Sz isolated strains and Population 3 (**B**).

**C,D.** Growth profile (**C**) and fumarate production for evolved Producer_F isolated strains and Population 3 (**D**).

**E,F.** Growth profile (**E**) and malate production for evolved Producer_Mz isolated strains and Population 2 (**F**).

Data information: Target compounds titer was evaluated at the end of growth curve (Max $OD_{600}$). The $OD_{600}$ (**A**, **C** and **E**) was measured during cultivation for the 9 isolated colonies, one population and the respective parental strain without or with glycine supplementation ($^{+gly}$). Populations (1,2 or 3) were plated in solid minimal media with 20 g $L^{-1}$ of glucose and three isolated colonies (named S, M or L) were selected for characterization.

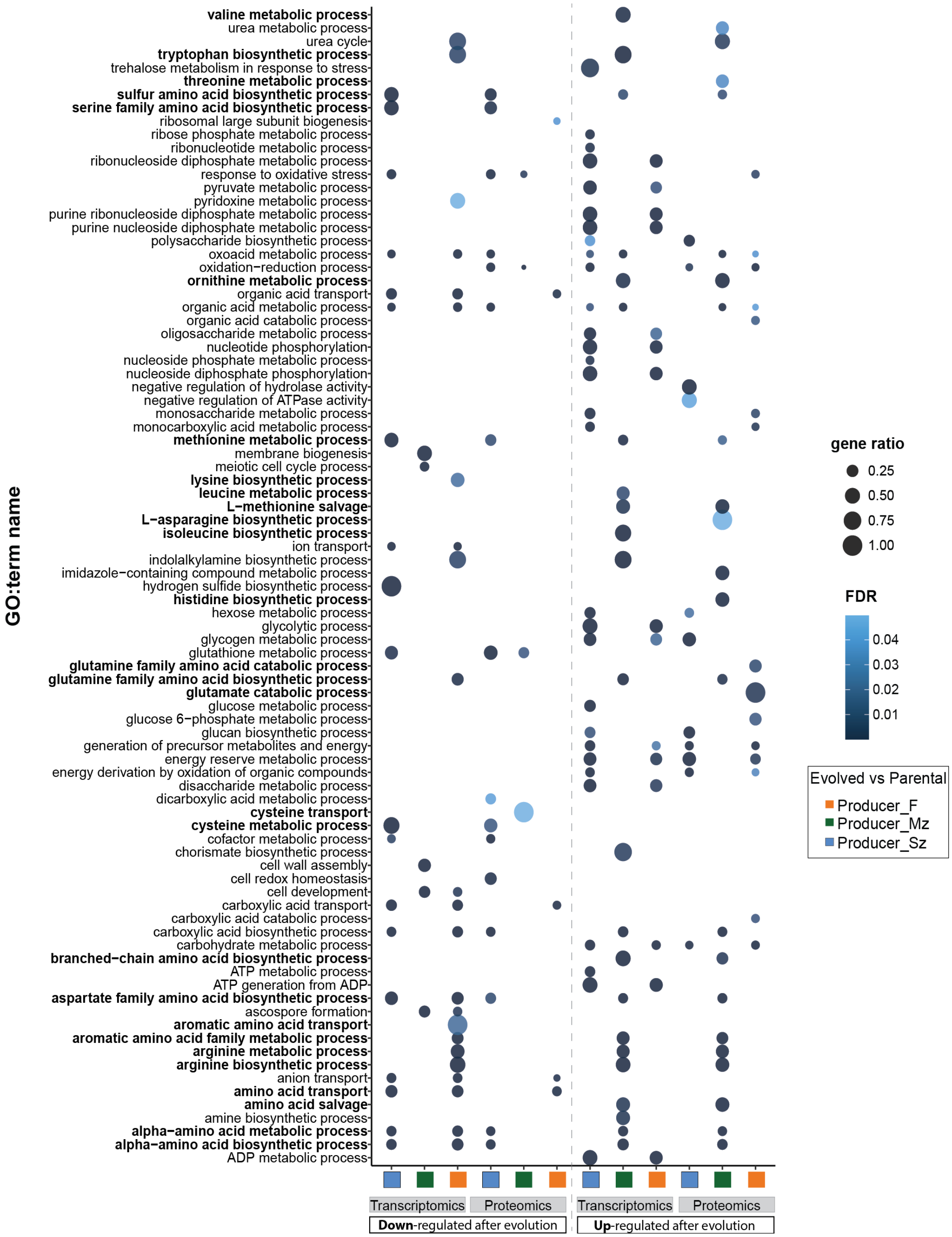


**Appendix Figure S4. GO term analyses of differentially expressed genes (Transcriptomics) and protein abundances (Proteomics) between evolved and parental strains.**

GO term enrichment analysis performed with gProfiler in RStudio. Dot colour (false discovery rate, FDR) and size (number of genes in query/number of genes associated to given GO:term, gene ratio). GO:term names highlighted in bold are related with amino acid metabolism.

**Appendix Table S1.** List of modifications to the original iMM904 model.

| Modification | Details | Source |
|---|---|---|
| Added reaction r_4039 | acetate_m + succinyl_CoA_m<---->succinate_m + acetyl_CoA_m | Yeast 7.11 |
| Modified r_0718 | NADP_m + S_malate_m----->NADPH_m + pyruvate_m + carbon_dioxide_m | Yeast 7.11 |
| Modified r_0773 | NADH_m + H_m + ubiquinone_6_m-----> NAD_m + ubiquinol_6_m | Yeast 7.11 |
| Modified r_0226 | 4 H_c + 1 ADP_m + 1 phosphate_m -----> 3 H_m + 1 H2O_m + 1 ATP_m | Yeast 7.11 |
| Modified r_2129 | H_m <----- H_c | Yeast 7.11 |
| Modified r_1110 | ADP_c + ATP_m <----> ATP_c + ADP_m | Yeast 7.11 |
| Modified r_0470 | NAD_c + H2O_c + L_glutamate_c -----> H_c + NADH_c + ammonium_c + 2_oxoglutarate_c | Yeast 7.11 |
| Removed r_0303 | citrate_c -----> H2O + cis_aconitate_c | Yeast 7.11 |
| Removed r_0338 | ubiquinone_6_m + S_dihydroorotate_c -----> ubiquinol_6_m + orotate_c | Yeast 7.11 |
| Removed r_0339 | oxygen_c + S_dihydroorotate_c -----> hydrogen_peroxide_c + orotate_c | Yeast 7.11 |
| Removed r_2127 | NAD_c + S_dihydroorotate_c -----> NADH_c + orotate_c | Yeast 7.11 |
| Modified r_1254 | H_e + pyruvate_e <----> H_c + pyruvate_c | Yeast 7.11 |
| Gene rule update r_0530 | (YPL252C /YDR376W) or (YPL252C/ YDR376W/ YER141W) | Yeast 7.11 |
| Updated biomass reaction r_4041 | Add 1.0E-6 * chitin_c and 1.0E-6* heme_a_m to reactants | Yeast 7.11 |
| Gene rule update r_0076 | (YER037W or YGL224C) | Yeast 7.11 |
| Gene rule update r_0078 | (YER037W or YGL224C) | Yeast 7.11 |
| Gene rule update r_1619 | YER037W | Yeast 7.11 |
| Removed r_0333 | phosphate_c + 2_deoxyuridine_c <----> uracil_c + 2_deoxy_D_ribofuranose_1_phosphate_c | Yeast 7.11 |
| Removed r_0944 | phosphate_c + adenosine_c<----> alpha_D_ribose_1_phosphate_c + adenine_c | Yeast 7.11 |
| Removed r_0945 | phosphate_m + adenosine_m <----> adenine_m + alpha_D_ribose_1_phosphate_m | Yeast 7.11 |
| Removed r_0946 | phosphate_c + 2_deoxyadenosine_c <----> 2_deoxy_D_ribofuranose_1_phosphate_c + adenine_c | Yeast 7.11 |
| Removed r_0947 | phosphate_c + 2_deoxyguanosine_c <----> guanine_c + 2_deoxy_D_ribofuranose_1_phosphate_c | Yeast 7.11 |
| Removed r_0948 | phosphate_c + 2_deoxyinosine_c <----> hypoxanthine_c + 2_deoxy_D_ribofuranose_1_phosphate_c | Yeast 7.11 |
| Removed r_0952 | phosphate_c + xanthosine_c <----> alpha_D_ribose_1_phosphate_c + 9H_xanthine_c | Yeast 7.11 |
| Removed r_1044 | phosphate_c + thymidine_c <----> 2_deoxy_D_ribofuranose_1_phosphate_c + thymine_c | Yeast 7.11 |
| Added reaction r_4045 | H2O_c + uridine_c -----> uracil_c + D_ribose_c | Yeast 7.11 |
| Gene rule update r_0888 | (YMR105C or YKL127W) | Yeast 7.11 |
| Gene rule update r_0907 | (YMR278W or YMR105C) or YKL127W) | Yeast 7.11 |
| Modified r_0110 | H_c + coenzyme_A_c + acetate_c <----- H2O_c + acetyl_CoA_c | Pereira et al., 2016 |
| Added reaction r_0234x | NAD_c + zymosterol_intermediate_1c_c -----> H_c + NADH_c + carbon_dioxide_c + zymosterol_intermediate_2_c | Pereira et al., 2016 |
| Added reaction r_0939x | prephenate_c + NAD_c -----> NADH_c + 3_4_hydroxyphenylpyruvate_c + carbon_dioxide_c | Pereira et al., 2016 |
| Modified r_1117 | H_c + L_aspartate_c -----> H_m + L_aspartate_m | Pereira et al., 2016 |
| Inactivated r_1840 | 3_hydroxy_3_methylglutaryl_CoA_c <-----> 3_hydroxy_3_methylglutaryl_CoA_m | No data supporting this reaction |

**Appendix Table S2**. Contribution of each gene knockout for succinate overproduction.

| **Gene Deletion(s)** | **Inactivated reaction(s)** | Biomass rate [1] | Succinate Flux [2] | **BPCY** [3] | **CYIELD** [4] | **Product Yield** ($Y_{P/S}$) [5] | **Biomass Yield** ($Y_{X/S}$) [6] |
|---|---|---|---|---|---|---|---|
| *Wild-type* | - | 0.1062 | 0.00000 | 0.00000 | 0.00000 | 0.00000 | 0.51266 |
| *SDH3* | R_SUCD1m; R_SUCD2_u6m; R_SUCD3_u6m | 0.0950 | 0.00006 | 0.00000 | 0.00003 | 0.00002 | 0.45853 |
| *SDH3+SER3* | R_SUCD1m; R_SUCD2_u6m; R_SUCD3_u6m | 0.0950 | 0.00006 | 0.00000 | 0.00003 | 0.00002 | 0.45853 |
| *SDH3+SER33* | R_SUCD1m; R_SUCD2_u6m; R_SUCD3_u6m | 0.0950 | 0.00006 | 0.00000 | 0.00003 | 0.00002 | 0.45853 |
| *SDH3+MET2* | R_SUCD1m; R_SUCD2_u6m; R_SUCD3_u6m; R_HSERTA | 0.0950 | 0.00006 | 0.00000 | 0.00003 | 0.00002 | 0.45853 |
| *SDH3+ZWF1* | R_SUCD1m; R_SUCD2_u6m; R_SUCD3_u6m; R_G6PDH2 | 0.0621 | 0.75267 | 0.04064 | 0.43633 | 0.28600 | 0.29973 |
| *SDH3+SER3+MET2* | R_SUCD1m; R_SUCD2_u6m; R_SUCD3_u6m; R_HSERTA | 0.0950 | 0.00006 | 0.00000 | 0.00003 | 0.00002 | 0.45853 |
| *SDH3+SER33+MET2* | R_SUCD1m; R_SUCD2_u6m; R_SUCD3_u6m; R_HSERTA | 0.0950 | 0.00006 | 0.00000 | 0.00003 | 0.00002 | 0.45853 |
| *SDH3+SER3+SER33* | R_SUCD1m; R_SUCD2_u6m; R_SUCD3_u6m; R_PGCD | 0.0918 | 0.00015 | 0.00001 | 0.00009 | 0.00006 | 0.44308 |
| *SDH3+ZWF1+SER3* | R_SUCD1m; R_SUCD2_u6m; R_SUCD3_u6m; R_G6PDH2 | 0.0621 | 0.75267 | 0.04064 | 0.43633 | 0.28600 | 0.29973 |
| *SDH3+ZWF1+SER33* | R_SUCD1m; R_SUCD2_u6m; R_SUCD3_u6m; R_G6PDH2 | 0.0621 | 0.75267 | 0.04064 | 0.43633 | 0.28600 | 0.29973 |
| *SDH3+ZWF1+MET2* | R_SUCD1m; R_SUCD2_u6m; R_SUCD3_u6m; R_G6PDH2; R_HSERTA | 0.0620 | 0.75307 | 0.04060 | 0.43656 | 0.28615 | 0.29925 |
| *SDH3+SER3+SER33+ MET2* | R_SUCD1m; R_SUCD2_u6m; R_SUCD3_u6m; R_PGCD; R_HSERTA | 0.0914 | 0.00015 | 0.00001 | 0.00009 | 0.00006 | 0.44115 |
| *SDH3+SER3+MET2+ ZWF1* | R_SUCD1m; R_SUCD2_u6m; R_SUCD3_u6m; R_G6PDH2; R_HSERTA | 0.0620 | 0.75307 | 0.04060 | 0.43656 | 0.28615 | 0.29925 |
| *SDH3+SER33+MET2+ ZWF1* | R_SUCD1m; R_SUCD2_u6m; R_SUCD3_u6m; R_G6PDH2; R_HSERTA | 0.0620 | 0.75307 | 0.04060 | 0.43656 | 0.28615 | 0.29925 |
| *SDH3+SER33+SER33+ ZWF1* | R_SUCD1m; R_SUCD2_u6m; R_SUCD3_u6m; R_PGCD; R_G6PDH2 | 0.0608 | 0.77816 | 0.04114 | 0.45111 | 0.29569 | 0.29346 |
| *SDH3+SER3+SER33+ MET2+ZWF1* | R_SUCD1m; R_SUCD2_u6m; R_SUCD3_u6m; R_PGCD; R_G6PDH2; R_HSERTA | 0.0606 | 0.78075 | 0.04114 | 0.45261 | 0.29667 | 0.29249 |

[1] $h^{-1}$

[2] $mmol.gCDW^{-1}.h^{-1}$

[3] mol succinate. mol $glucose^{-1}.h^{-1}$

[4] C-mol succinate. C-mol $glucose^{-1}$

[5] g succinate. g $glucose^{-1}$

[6] g CDW. g glucose

**Appendix Table S3.** Contribution of each gene knockout for fumarate overproduction.

| **Gene Deletion(s)** | **Inactivated reaction(s)** | Biomass rate [1] | Fumarate Flux [2] | **BPCY** [3] | **CYIELD** [4] | **Product Yield** ($Y_{P/S}$) [5] | **Biomass Yield** ($Y_{X/S}$) [6] |
|---|---|---|---|---|---|---|---|
| Wild-type | - | 0.1062 | 0.00000 | 0.00000 | 0.00000 | 0.00000 | 0.51266 |
| *FUM1* | R_FUM; R_FUMm | 0.0936 | 0.03527 | 0.00287 | 0.02045 | 0.01317 | 0.45177 |
| *FUM1+SER3* | R_FUM; R_FUMm | 0.0936 | 0.03527 | 0.00287 | 0.02045 | 0.01317 | 0.45177 |
| *FUM1+SER33* | R_FUM; R_FUMm | 0.0936 | 0.03527 | 0.00287 | 0.02045 | 0.01317 | 0.45177 |
| *FUM1+MET2* | R_FUM; R_FUMm; R_HSERTA | 0.0935 | 0.03525 | 0.00287 | 0.02043 | 0.01317 | 0.45129 |
| *FUM1+ZWF1* | R_FUM; R_FUMm; R_G6PDH2 | 0.0686 | 0.72404 | 0.04319 | 0.41973 | 0.27042 | 0.33111 |
| *FUM1+SER3+MET2* | R_FUM; R_FUMm; R_HSERTA | 0.0935 | 0.03525 | 0.00287 | 0.02043 | 0.01317 | 0.45129 |
| *FUM1+SER33+MET2* | R_FUM; R_FUMm; R_HSERTA | 0.0935 | 0.03525 | 0.00287 | 0.02043 | 0.01317 | 0.45129 |
| *FUM1+SER3+SER33* | R_FUM; R_FUMm; R_PGCD | 0.0906 | 0.10056 | 0.00792 | 0.05830 | 0.03756 | 0.43729 |
| *FUM1+ZWF1+SER3* | R_FUM; R_FUMm; R_G6PDH2 | 0.0690 | 0.7175 | 0.04305 | 0.41594 | 0.26798 | 0.33304 |
| *FUM1+ZWF1+SER33* | R_FUM; R_FUMm; R_G6PDH2 | 0.0690 | 0.7175 | 0.04305 | 0.41594 | 0.26798 | 0.33304 |
| *FUM1+ZWF1+MET2* | R_FUM; R_FUMm; R_G6PDH2; R_HSERTA | 0.0686 | 0.72444 | 0.04321 | 0.41997 | 0.27057 | 0.33111 |
| *FUM1+SER3+SER33+MET2* | R_FUM; R_FUMm; R_PGCD; R_HSERTA | 0.0902 | 0.10932 | 0.00857 | 0.06337 | 0.04083 | 0.43536 |
| *FUM1+SER3+MET2+ZWF1* | R_FUM; R_FUMm; R_G6PDH2; R_HSERTA | 0.0686 | 0.72444 | 0.04321 | 0.41997 | 0.27057 | 0.33111 |
| *FUM1+SER33+MET2+ZWF1* | R_FUM; R_FUMm; R_G6PDH2; R_HSERTA | 0.0686 | 0.72444 | 0.04321 | 0.41997 | 0.27057 | 0.33111 |
| *FUM1+SER33+SER33+ZWF1* | R_FUM; R_FUMm; R_PGCD; R_G6PDH2 | 0.0675 | 0.75026 | 0.04404 | 0.43493 | 0.28021 | 0.32580 |
| *FUM1+SER3+SER33+MET2+ ZWF1* | R_FUM; R_FUMm; R_PGCD; R_G6PDH2; R_HSERTA | 0.0673 | 0.75292 | 0.04406 | 0.43648 | 0.28120 | 0.32483 |

[1] $h^{-1}$

[2] $mmol.gCDW^{-1}.h^{-1}$

[3] mol fumarate. mol $glucose^{-1}.h^{-1}$

[4] C-mol fumarate. C-mol $glucose^{-1}$

[5] g fumarate. g $glucose^{-1}$

[6] g CDW. g $glucose^{-1}$

**Appendix Table S4.** Contribution of each gene knockout for malate overproduction.

| Gene Deletion(s) | Inactivated reaction(s) | Biomass rate[1] | Malate Flux[2] | **BPCY** [3] | **CYIELD** [4] | **Product Yield** ($Y_{P/S}$) [5] | **Biomass Yield** ($Y_{X/S}$) [6] |
|---|---|---|---|---|---|---|---|
| Wild-type | - | 0.1062 | 0.00000 | 0.0000 | 0.0000 | 0.00000 | 0.51266 |
| MSK (*MDH1+MDH2+ MAE1*) | R_ME2m; R_ME1m; R_MDHm; R_MDH | 0.0932 | 0.03507 | 0.0028 | 0.0203 | 0.01513 | 0.44984 |
| MSK+*SER3* | R_ME2m; R_ME1m; R_MDHm; R_MDH | 0.0932 | 0.03507 | 0.0028 | 0.0203 | 0.01513 | 0.44984 |
| MSK+*SER33* | R_ME2m; R_ME1m; R_MDHm; R_MDH | 0.0932 | 0.03507 | 0.0028 | 0.0203 | 0.01513 | 0.44984 |
| MSK+*MET2* | R_ME2m; R_ME1m; R_MDHm; R_MDH; R_HSERTA | 0.0931 | 0.03505 | 0.0028 | 0.0203 | 0.01512 | 0.44936 |
| MSK+*IRC7* | R_ME2m; R_ME1m; R_MDHm; R_MDH; R_THRAi; R_THRA2i | 0.0932 | 0.03507 | 0.0028 | 0.0203 | 0.01513 | 0.44984 |
| MSK+*ZWF1* | R_ME2m; R_ME1m; R_MDHm; R_MDH; R_G6PDH2 | 0.0619 | 0.76549 | 0.0412 | 0.4438 | 0.33028 | 0.29877 |
| MSK+*MET2+SER3* | R_ME2m; R_ME1m; R_MDHm; R_MDH; R_HSERTA | 0.0931 | 0.03505 | 0.0028 | 0.0203 | 0.01512 | 0.44936 |
| MSK+*MET2+SER33* | R_ME2m; R_ME1m; R_MDHm; R_MDH; R_HSERTA | 0.0931 | 0.03505 | 0.0028 | 0.0203 | 0.01512 | 0.44936 |
| MSK+*SER3+GLY1* | R_ME2m; R_ME1m; R_MDHm; R_MDH; R_THRAi; R_THRA2i | 0.0932 | 0.03985 | 0.0032 | 0.0231 | 0.01719 | 0.44984 |
| MSK+*SER33+IRC7* | R_ME2m; R_ME1m; R_MDHm; R_MDH; R_THRAi; R_THRA2i | 0.0932 | 0.03507 | 0.0028 | 0.0203 | 0.01513 | 0.44984 |
| MSK+*MET2+IRC7* | R_ME2m; R_ME1m; R_MDHm; R_MDH; R_HSERTA; R_THRAi; R_THRA2i | 0.0932 | 0.03507 | 0.0028 | 0.0203 | 0.01513 | 0.44984 |
| MSK+*MET2+ZWF1* | R_ME2m; R_ME1m; R_MDHm; R_MDH; R_HSERTA; R_G6PDH2 | 0.0619 | 0.76587 | 0.0412 | 0.4440 | 0.33045 | 0.29877 |
| MSK+*ZWF1+SER3* | R_ME2m; R_ME1m; R_MDHm; R_MDH; R_G6PDH2 | 0.0619 | 0.76549 | 0.0412 | 0.4438 | 0.33028 | 0.29877 |
| MSK+*ZWF1+SER33* | R_ME2m; R_ME1m; R_MDHm; R_MDH; R_G6PDH2 | 0.0619 | 0.76549 | 0.0412 | 0.4438 | 0.33028 | 0.29877 |
| MSK+*SER3+SER33* | R_ME2m; R_ME1m; R_MDHm; R_MDH; R_PGCD | 0.0903 | 0.03445 | 0.0027 | 0.0200 | 0.01486 | 0.43584 |
| MSK+*SER3+SER33+MET2* | R_ME2m; R_ME1m; R_MDHm; R_MDH; R_PGCD; R_HSERTA | 0.0899 | 0.03431 | 0.0027 | 0.0199 | 0.01480 | 0.43391 |
| MSK+*SER33+SER3+ZWF1* | R_ME2m; R_ME1m; R_MDHm; R_MDH; R_PGCD; R_G6PDH2 | 0.0602 | 0.79314 | 0.0415 | 0.4598 | 0.34221 | 0.29056 |
| MSK+*SER33+SER3+IRC7* | R_ME2m; R_ME1m; R_MDHm; R_MDH; R_PGCD; R_THRAi; R_THRA2i | 0.0903 | 0.03445 | 0.0027 | 0.0200 | 0.01486 | 0.43584 |
| MSK+*SER3+MET2+IRC7* | R_ME2m; R_ME1m; R_MDHm; R_MDH; R_HSERTA; R_THRAi; R_THRA2i | 0.0927 | 0.04426 | 0.0036 | 0.0257 | 0.01910 | 0.44743 |
| MSK+*SER33+MET2+IRC7* | R_ME2m; R_ME1m; R_MDHm; R_MDH; R_HSERTA; R_THRAi; R_THRA2i | 0.0927 | 0.04426 | 0.0036 | 0.0257 | 0.01910 | 0.44743 |
| MSK+*MET2+ZWF1+IRC7* | R_ME2m; R_ME1m; R_MDHm; R_MDH; R_HSERTA; R_G6PDH2; R_THRAi; R_THRA2i | 0.0616 | 0.7708 | 0.0413 | 0.4468 | 0.33258 | 0.29732 |
| MSK+*SER33+SER3+ IRC7+MET2* | R_ME2m; R_ME1m; R_MDHm; R_MDH; R_PGCD; R_THRAi; R_THRA2i; R_HSERTA | 0.0895 | 0.04321 | 0.0034 | 0.0250 | 0.01864 | 0.43198 |
| MSK+*SER33+SER33+ IRC7+ZWF1* | R_ME2m; R_ME1m; R_MDHm; R_MDH; R_PGCD; R_THRAi; R_THRA2i; R_G6PDH2 | 0.0602 | 0.79314 | 0.0415 | 0.4598 | 0.34221 | 0.29056 |
| MSK+*SER33+SER33+ IRC7+MET2+ZWF1* | R_ME2m; R_ME1m; R_MDHm; R_MDH; R_PGCD; R_THRAi; R_THRA2i; R_HSERTA; R_G6PDH2 | 0.0597 | 0.8005 | 0.0416 | 0.4641 | 0.34539 | 0.28815 |

[1] $h^{-1}$

[2] $mmol.gCDW^{-1}.h^{-1}$

[3] mol fumarate. mol $glucose^{-1}.h^{-1}$

[4] C-mol fumarate. C-mol $glucose^{-1}$

[5] g fumarate. g $glucose^{-1}$

[6] g CDW. g $glucose^{-1}$

**Appendix Table S5.** Impact of each gene deletion in the selected solutions using LMOMA and the iMM904 model.

| Target compounds | Gene Deletion(s) | Biomass rate[1] | Target Flux[2] | BPCY[3] | CYIELD[4] |
|---|---|---|---|---|---|
| **Succinate** | SDH-complex * | 0.00792 | 0.96589 | 0.00665 | 0.55994 |
| | SDH-c+*ZWF1* | 0.00000 | 1.01621 | 0.00000 | 0.58911 |
| | SDH-c+*SER3*+*SER33* | 0.00424 | 1.00873 | 0.00372 | 0.58477 |
| | SDH-c+*SER3*+*SER33*+*ZWF1* | 0.00000 | 1.09639 | 0.00000 | 0.63559 |
| **Fumarate** | FUM1 | 0.03327 | 0.87633 | 0.02535 | 0.50802 |
| | FUM1+*ZWF1* | 0.02894 | 0.92834 | 0.02336 | 0.53817 |
| | FUM1+*SER3*+*SER33* | 0.03458 | 0.86465 | 0.02600 | 0.50125 |
| | FUM1+*SER3*+*SER33*+*ZWF1* | 0.02684 | 0.95174 | 0.02221 | 0.55173 |
| **Malate** | MSK ** | 0.01910 | 0.93368 | 0.01551 | 0.54126 |
| | MSK+*ZWF1* | 0.01050 | 0.12954 | 0.00118 | 0.07510 |
| | MSK+*SER3*+*SER33* | 0.01687 | 0.83475 | 0.01225 | 0.48391 |
| | MSK+*SER3*+*SER33*+*ZWF1* | 0.00788 | 0.98084 | 0.00672 | 0.56860 |
| | Wild-type | 0.10622 | 0.00000 | 0.00000 | 0.00000 |

[1] $h^{-1}$

[2] $mmol.gCDW^{-1}.h^{-1}$

[3] mol target product. mol $glucose^{-1}.h^{-1}$

[4] C-mol target product. C-mol $glucose^{-1}$

* SDH-c = SDH-complex

* MSK = MDH1+MDH2+MAE1

**Appendix Table S6.** Analysis of the *in-silico* productivity values using different *S. cerevisiae* GSMMs and simulation methods. Simulations were performed in Optflux v3.2.8, using CPLEX ILOG solver, with a glucose uptake rate of 1.15 $mmol.gCDW.h^{-1}$.

| | | | | CYIELD[1] | | BPCY[2] | |
|---|---|---|---|---|---|---|---|
| Target compounds | Gene deletions | | GSMM | pFBA | LMOMA | pFBA | LMOMA |
| Fumarate | *SER 3* | *FUM1* | iMM904* | 0.4349 | 0.5517 | 0.044 | 0.0222 |
| | *SER 33* | | iND750 | 0.4210 | 0.5331 | 0.0452 | 0.0209 |
| | *ZWF1* | | Yeast 6* | 0.4657 | 0.584 | 0.0348 | 0.0069 |
| Succinate | *SER 3* | *SDH2* | iMM904* | 0.4511 | 0.6356 | 0.0411 | 0.0000 |
| | *SER 33* | | iND750 | 0.0603 | 0.5206 | 0.006 | 0.0000 |
| | *ZWF1* | | Yeast 6* | 0.0411 | 0.6021 | 0.003 | 0.0000 |
| Malate | *SER3* | *MAE1* | iMM904* | 0.4598 | 0.5686 | 0.0415 | 0.0067 |
| | *SER33* | *MDH1* | iND750 | 0.421 | 0.0452 | 0.0236 | 0.0009 |
| | *ZWF1* | *MDH2* | Yeast 6* | 0.0098 | 0.1885 | 0.0007 | 0.0000 |

[1] C-mol target product. C-mol $glucose^{-1}.h^{-1}$

[2] mol target product. mol $glucose^{-1}.h^{-1}$

* Modified versions (see Supplementary Table 1)

## **Appendix Table S7.** *Saccharomyces cerevisiae* strains used in this study.

| Strain name[a] | Genotype | Details | Source |
|---|---|---|---|
| CEN.PK119 | *MATa/MATalfa URA3/ura3-52 MAL2-* 8c/ *MAL2-* 8c SUC2/ SUC2 | (diploid wild-type) | P. Kötter[b] |
| **WT (CEN.PK113-7D)** | *MATa URA3 MAL2-* $8^{c}$ *SUC2* | (wild-type, reference strain) | P. Kötter[b] |
| **Chassis** | MATa *URA3 MAL2-* 8c *SUC2* ser3Δ ser33Δ | ser3(1,1240)::loxP-**kanMX4**-loxP<br>ser33(1,1090)::loxP-**natMX4**-loxP | This study |
| **Chassis_z** | *MATa URA3 MAL2-* $8^{c}$ *SUC2 ser3Δ ser33Δ zwf1Δ* | ser33(1,1090)::loxP-**natMX4**-loxP<br>ser3(1,1240)::loxP-**kanMX4**-loxP<br>zwf1(41,1475)::loxP-**kanMX4**-loxP | This study |
| **S1** | MATa URA3 MAL2- $8^{c}$ SUC2 *sdh3Δ* | sdh3(1,540)::loxP-**hphMX4**-loxP | This study |
| **Producer_S** | MATa URA3 MAL2-$8^{c}$ SUC2 *ser3Δ ser33Δ sdh3Δ* | ser3(1,1240)::loxP-**kanMX4**-loxP<br>ser33(1,1090)::loxP-**natMX4**-loxP<br>sdh3(1,540)::loxP-**hphMX4**-loxP | This study |
| **Producer_Sz** | MATa URA3 MAL2- 8c SUC2 *ser3Δ ser33Δ sdh3Δ zwf1Δ* | ser33(1,1090)::loxP-**natMX4**-loxP<br>sdh3(1,540)::loxP-**hphMX4**-loxP<br>ser3(1,1240)::loxP-**kanMX4**-loxP<br>zwf1(41,1475)::loxP-**kanMX4**-loxP | This study |
| **M1** | MATa URA3 MAL2- 8c SUC2 *mdh1Δ mdh2Δ mae1Δ* | mdh1(1,900)::loxP-**natMX4**-loxP<br>mdh2(41,1259)::loxP-**hphMX4**-loxP<br>mae1(41,1269)::loxP-**KanMX**-loxP | This study |
| **Producer_M** | MATa URA3 MAL2- 8c SUC2 *ser3Δ ser33Δ mdh1Δ mdh2Δ mae1Δ* | ser3(1,1240)::loxP-**kanMX4**-loxP<br>ser33(1,1090)::loxP-**natMX4**-loxP<br>mdh1(1,900)::loxP-**natMX4**-loxP<br>mdh2(41,1094)::loxP-**hphMX4**-loxP<br>mae1(41,1269)::loxP-**hphMX4**-loxP | This study |
| **Producer_Mz** | *MATa URA3 MAL2-* $8^{c}$ *SUC2 ser3Δ ser33Δ zwf1Δ mdh1Δ mdh2Δ mae1Δ* | zwf1(41,1475)::loxP-**kanMX4**-loxP<br>ser3(1,1240)::loxP-**kanMX4**-loxP<br>ser33(1,1090)::loxP-**natMX4**-loxP<br>mdh1(1,900)::loxP-**natMX4**-loxP<br>mdh2(41,1094)::loxP-**hphMX4**-loxP<br>mae1(41,1269)::loxP-**hphMX4**-loxP | This study |
| **F1** | MATa URA3 MAL2- 8c SUC2 *fum1Δ* | fum1(1,1050)::loxP-**hphMX4**-loxP | This study |
| **Producer_F** | MATa URA3 MAL2- 8c SUC2 *ser3Δ ser33Δ fum1Δ* | ser33(1,1090)::loxP-**natMX4**-loxP<br>ser3(1,1240)::loxP-**kanMX4**-loxP<br>fum1(1,1050)::loxP-**hphMX4**-loxP | This study |
| **Producer_Fz** | MATa URA3 MAL2- 8c SUC2 *ser3Δ ser33Δ zwf1Δ fum1Δ* | fum1(1,1050)::loxP-**hphMX4**-loxP<br>zwf1(41,1475)::loxP-**hphMX4**-loxP<br>ser3(1,1240)::loxP-**kanMX4**-loxP<br>ser33(1,1090)::loxP-**natMX4**-loxP | This study |
| **Producer_FzG** | MATa *ura3-52* MAL2- 8c SUC2 *ser3Δ ser33Δ zwf1Δ fum1Δ gdh1Δ::GDH2* | fum1(1,1050)::loxP-**hphMX4**-loxP<br>zwf1(41,1475)::loxP-**hphMX4**-loxP<br>ser3(1,1240)::loxP-**kanMX4**-loxP<br>ser33(1,1090)::loxP-**natMX4**-loxP<br>gdh1::GDH2_**URA3MX4** | This study |
| **E_Chassis_z** | *MATa URA3 MAL2-* $8^{c}$ *SUC2 ser3Δ ser33Δ zwf1Δ* | Evolved isolate from Chassis_z, | This study |
| **E_Producer_Sz** | MATa URA3 MAL2- 8c SUC2 *ser3Δ ser33Δ sdh3Δ zwf1Δ* | Evolved isolate from Producer_Sz,<br>(isolate SZ-3-M) | This study |
| **E_Producer_F** | MATa URA3 MAL2- 8c SUC2 *ser3Δ ser33Δ sdh3Δ zwf1Δ* | Evolved isolate from Producer_F,<br>(isolate F-2-M) | This study |
| **E_Producer_Mz** | MATa URA3 MAL2- 8c SUC2 *ser3Δ ser33Δ sdh3Δ zwf1Δ* | Evolved isolate from Producer_Mz,<br>(isolate MZ-2-L) | This study |
| **E_Producer_Fz** | MATa URA3 MAL2- 8c SUC2 *ser3Δ ser33Δ sdh3Δ zwf1Δ* | Evolved isolate from Producer_Fz,<br>(isolate 2) | This study |
| **E_Producer_FzG** | MATa URA3 MAL2- 8c SUC2 *ser3Δ ser33Δ sdh3Δ zwf1Δ* | Evolved isolate from Producer_FzG,<br>(isolate 3) | This study |

[a] Strain name used in the results section, where the letter “S” for succinate, “F” for fumarate, “M” for malate, “z” indicates that *ZWF1* gene is deleted in the chassis strain genotype and “E_” stands for evolved strain.
[b] Institut für Mikrobiologie, der Johan Wolfgang Goethe-Universität, Frankfurt am Main, Germany (EUROSCARF).

**Appendix Table S8.** List of primers used in this study

| Name | Sequence 5'→ 3' |
|---|---|
| **Primers for deletion-cassette amplification** | |
| SDH3-F1 | ACCAAAAATTGCCAATCACAAGCTCTAAGAATAATAAACG**CAGCTGAAGCTTCGTACGC** |
| SDH3-R1 | AAGTACCGAGAACGGCGGTGAAACCAATGAGAGCGTAACC**GCATAGGCCACTAGTGGATCTG** |
| FUM1-F1 | TCCATAAAGTCTAACTATTAAACGGATAAGAGATACAATC**CAGCTGAAGCTTCGTACGC** |
| FUM1-R1 | AGATTTAGTCAACAACTCATGAATACGAGGCTCATTGGCT**GCATAGGCCACTAGTGGATCTG** |
| SER3-F1 | ATACAGAACTCTATAAAGAACCACAGAAAAATCGACAGCA**CAGCTGAAGCTTCGTACGC** |
| SER3-R1 | ATTGCTTTTCGATGTTATGGTTCGATAAAATATCATTGA**GCATAGGCCACTAGTGGATCTG** |
| SER33-F1 | AAAAGTAACAAACACTGATTTCGGGTATTTCCTCCCTAAC**CAGCTGAAGCTTCGTACGC** |
| SER33-R1 | ACAGAGTTACCTTCATTGATGTATTTGGACAATGCAGTAG**GCATAGGCCACTAGTGGATCTG** |
| ZWF1-S1 | ATGAGTGAAGGCCCCGTCAAATTCGAAAAAAATACCGTCA**CAGCTGAAGCTTCGTACGC** |
| ZWF1-S2 | ATTATCCTTCGTATCTTCTGGCTTAGTCACGGGCCAAGCG**GCATAGGCCACTAGTGGATCTG** |
| MAE1-S1 | ATGCTTAGAACCAGACTATCCGTTTCCGTTGCTGCTAGAT**CAGCTGAAGCTTCGTACGC** |
| MAE1-S2 | GGGCGGGAGTGGAGTTAGCCTCGTAAGATTGTAGAATTAA**GCATAGGCCACTAGTGGATCTG** |
| MDH1-F1 | AAGAAAAAAAACAAAAGGAAAAGGAAGGATACCATATACA**CAGCTGAAGCTTCGTACGC** |
| MDH1-R1 | GTAGCATTTCTTCTTCTTCTGAAGATAACTCACCTATTGG**GCATAGGCCACTAGTGGATCTG** |
| MDH2-S1 | ATGCCTCACTCAGTTACACCATCCATAGAACAAGATTCGT**CAGCTGAAGCTTCGTACGC** |
| MDH2-S2 | TTAAGATGATGCAGATCTCGATGCAACGAATTCCAAGCCC**GCATAGGCCACTAGTGGATCTG** |
| **Diagnostic primers for deletion confirmation** | |
| SDH3-A1 | GCTGTATACCAACAGCCTTC |
| SDH3-A4 | ATGACCGCCTATGTTTGC |
| FUM1-A1 | TCCTTAAACCCTTCCGAATC |
| FUM1-A4 | GTATGCCTATGCTCCTCTTC |
| SER3-A1 | TTAAGCAGTTAGGCTGGACC |
| SER3-A4 | AGAATTCGGGTTTGCGTTCC |
| SER33-A1 | TCGTTTATACTGGCTGACCC |
| SER33-A4 | AGCTCGACAGATTATCGTCC |
| ZWF1-A1 | ATCTGGTGCGTAAACTGACC |
| ZWF1-A4 | ATGGAGGGCAAAGGGACAG |
| MAE1-A1 | CATACAACCAAGTATAGACGG |
| MAE1-A6 | GTTGGTGATGGTGTCCAGG |
| MDH1-A5 | GTGCGCTGCAGGGTGCTAC |
| MDH1-A8 | CATTCAAAGCACGCATAGTAC |
| MDH2-A1 | GCCCTCTTCTGGCGCCTG |
| MDH2-A4 | CGTATTGCAGCGAGGGTTCC |
| K1-A | GGATGTATGGGCTAAATGTACG |
| K2-A | CATCATCTGCCCAGATGCG |
| MATa | ACTCCACTTCAAGTAAGAGTTTG |
| MATal | GCACGGAATATGGGACTACTTCG |
| MATa/al | AGTCACATCAAGATCGTTTATGG |

**Appendix Table S9.** Metabolites, retention time and ion fragments used for quantification with GC-MS

| **Metabolite** | **Metabolite_derivative** | **Retention Time (minutes)** | **Quantifying Ion Fragment (m/z)** |
|---|---|---|---|
| Lactic acid | Lactic acid-2TMS | 2.765 | 191.1 |
| Pyruvic acid | Pyruvic acid-meto-TMS | 3.742 | 174 |
| Glycerol | Glycerol-3TMS | 5.069 | 218.1 |
| Glycine | Glycine-3TMS | 6.112 | 174.1 |
| Serine | Serine-3TMS | 7.16 | 204.1 |
| Fumaric acid | Fumaric acid-2TMS | 7.562 | 245 |
| Succinic acid | Succinic acid-2TMS | 7.649 | 247.1 |
| Glyceric acid | Glyceric acid-3TMS | 8.081 | 189.1 |
| Malic acid | Malic acid-3TMS | 9.616 | 335 |
| 2-hydroxyglutaric acid | 2-Hydroxyglutaric acid-3TMS | 10.823 | 247.2 |
| Ribitol (*internal standard*) | Ribitol-5TMS | 10.892 | 319.1 |
| Putrescine | Putrescine-4TMS | 11.021 | 174.1 |
| 5-oxoproline | 5-Oxoproline-2TMS | 11.566 | 156.1 |
| 2-ketoglutaric acid | 2-Ketoglutaric acid-meto-2TMS | 12.01 | 198.1 |
| Ornithine | Ornithine-4TMS | 12.595 | 142.1 |
| Glucose | Glucose-meto-5TMS (peak 1) | 13.237 | 319.1 |
| Inositol | Inositol-6TMS (peak 1) | 14.623 | 305.1 |
| Palmitic acid | Palmitic acid-TMS | 15.605 | 313.3 |
| Stearic acid | Stearic acid-TMS | 17.338 | 341.3 |
| Oleic acid | Oleic acid-TMS | 17.355 | 339.3 |

**Appendix Table S10.** Production titers, yields and rates of the engineered strains, before and after evolution, from shake flask cultivations[a] and batch fermentations[b] .

| Target product | Strain | Growth rate $\mu_{max}$ ($h^{-1}$) | Max Titer (g $L^{-1}$) | Max Yield P/S (g. g-glc$^{-1}$) | Max Yield P/X (g. g biomass$^{-1}$) | Productivity (g L.$h^{-1}$) |
|---|---|---|---|---|---|---|
| **Succinate[a]** | WT | 0.332 | 0.018 | 0.0009 | 0.008 | 0.006 |
| | **Producer_Sz [+gly]** | **0.024** | **0.472** | **0.0455** | **0.614** | **0.012** |
| | **E_Producer_Sz** | **0.147** | **0.688** | **0.0344** | **0.504** | **0.101** |
| | Chassis_z | 0.122 | 0.033 | 0.0017 | 0.004 | 0.004 |
| | E_Chassis_z | 0.325 | 0.009 | 0.0005 | 0.017 | 0.003 |
| **Succinate[b]** *batch fermen-tation* | WT | 0.34 | 0.020 | 0.004 | 0.025 | 0.07 |
| | **Producer_Sz [+gly]** | **0.17** | **0.93** | **0.019** | **0.23** | **0.16** |
| | **E_Producer_Sz** | **0.17** | **1.12** | **0.022** | **0.28** | **0.19** |
| **Fumarate[a]** | WT | 0.332 | ND | ND | ND | ND |
| | **Producer_F [+gly]** | **0.039** | **0.253** | **0.023** | **0.317** | **0.010** |
| | **E_Producer_F** | **0.182** | **0.353** | **0.063** | **0.296** | **0.064** |
| | Chassis_z | 0.122 | 0.004[§] | 0.0002[§] | 0.002[§] | 0.000[§] |
| | E_Chassis_z | 0.325 | 0.002[§] | 0.0001[§] | 0.001[§] | 0.001[§] |
| **Malate[a]** | WT | 0.332 | 0.009 | 0.0004 | 0.004 | 0.003 |
| | **Producer_Mz [+gly]** | **0.088** | **0.468** | **0.0234** | **0.589** | **0.041** |
| | **E_Producer_Mz** | **0.127** | **0.255** | **0.0128** | **0.188** | **0.032** |
| | Chassis_z | 0.122 | 0.033 | 0.0016 | 0.016 | 0.004 |
| | E_Chassis_z | 0.325 | 0.004[§] | 0.0002[§] | 0.002[§] | 0.001[§] |

[a] Values are presented as means of biological triplicates grown in shake flasks in defined minimal medium (20 g $L^{-1}$ glucose). Standard deviation (s.d) for each sample was below 15%. [b] Values are presented as means of biological triplicates grown in controlled environment (batch fermenters) of samples taken in stationary phase. Glycine was supplemented with 0.5 g $L^{-1}$ , indicated by the suffix "+ gly". ND – compound not-detected; § - Not-quantified: Metabolite detected but AUC below quantification curve.

**Appendix Table S11.** Glucose consumption and secreted metabolite titers of WT and engineered strains, before and after evolution, from shake flask cultivations.

| Strains | Consumed | Secreted metabolites | | | | | | |
|---|---|---|---|---|---|---|---|---|
| | **Glucose** [a] | **Ethanol** [b] | **Pyruvate** [a] | **Acetate** [b] | **Lactate** [a] | **Succinate** [a] | **Fumarate** [a] | **Malate** [a] |
| WT | 19.984±0.001 | 8.009±0.001 | 0.048±0.000 | 2.202±0.000 | 0.014±0.004 | 0.018±0.005 | 0.002±0.000 | 0.008±0.001 |
| Chassis_z | 19.984±0.000 | 8.934±0.001 | 0.048±0.001 | 0.845±0.001 | 0.012±0.002 | 0.033±0.008 | 0.004±0.000 | 0.033±0.007 |
| E_Chassis_z | 19.984±0.000 | 7.410±0.001 | 0.049±0.000 | 3.503±0.002 | 0.027±0.005 | 0.009±0.000 | 0.002±0.000 | 0.004±0.000 |
| Producer_Sz* | 10.529±1.194 | 3.560±0.458 | 0.163±0.016 | 0.780±0.083 | 0.011±0.001 | **0.472±0.060** | 0.003±0.000 | 0.009±0.003 |
| E_ Producer_Sz | 19.984±0.000 | 6.510±0.046 | 0.087±0.057 | 3.360±0.114 | 0.016±0.001 | **0.688±0.088** | 0.002±0.000 | 0.005±0.001 |
| Producer_F* | 9.297±0.471 | 4.160±0.386 | 0.256±0.019 | 0.463±0.015 | 0.023±0.004 | 0.189±0.022 | **0.253±0.005** | 0.004±0.000 |
| E_Producer_F | 18.510±0.000 | 7.171±0.004 | 0.168±0.029 | 0.290±0.017 | 0.022±0.005 | 0.108±0.013 | **0.353±0.006** | 0.004±0.000 |
| Producer_Mz* | 14.666±2.315 | 3.558±0.001 | 0.163±0.061 | 0.778±0.001 | 0.014±0.002 | 0.031±0.002 | 0.039±0.005 | **0.468±0.020** |
| E_Producer_Mz | 19.984±0.000 | 6.510±0.000 | 0.052±0.001 | 3.356±0.01 | 0.016±0.006 | 0.019±0.004 | 0.016±0.003 | **0.255±0.045** |

Values are presented as average of 3 biological replicates grown in shake flasks in defined minimal medium (20 g $L^{-1}$ glucose) ± Standard deviation (s.d).

[a] Metabolite concentration was determined by GS-MS.

[b] Metabolite concentration was determined by UPLC.

The growth medium of parental strains was supplemented with glycine (0.5 g $L^{-1}$), indicated by '*'.
