# Supplementary material for: Model‐guided development of an evolutionarily stable yeast chassis
Source: Mol Syst Biol. 2021 Jul 22;17(7):e10253. doi: 10.15252/msb.202110253 (PMC8297383; doi:10.15252/msb.202110253)
Supplement: Supplementary file 2 — Expanded View Figures PDF [file MSB-17-e10253-s005.pdf]

Expanded View Figures

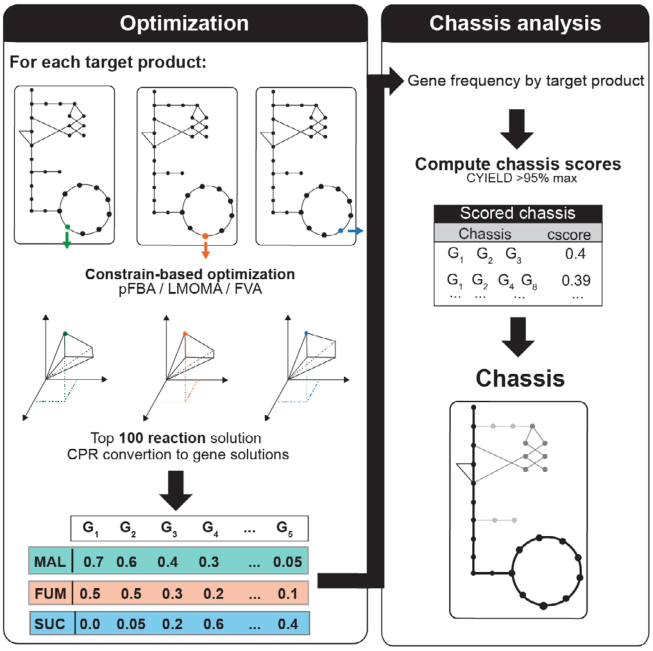

Figure EV1. Representation of chassis-strain *in silico* design framework.

The two main stages of the pipeline are depicted: (i) optimization stage, encompassing the generation of results, their post-processing and evaluation; and (ii) chassis analysis stage, which includes the gene frequency analysis, generation of candidate chassis, reconciliation with the generated solutions and scoring of the final chassis.

© 2021 The Authors

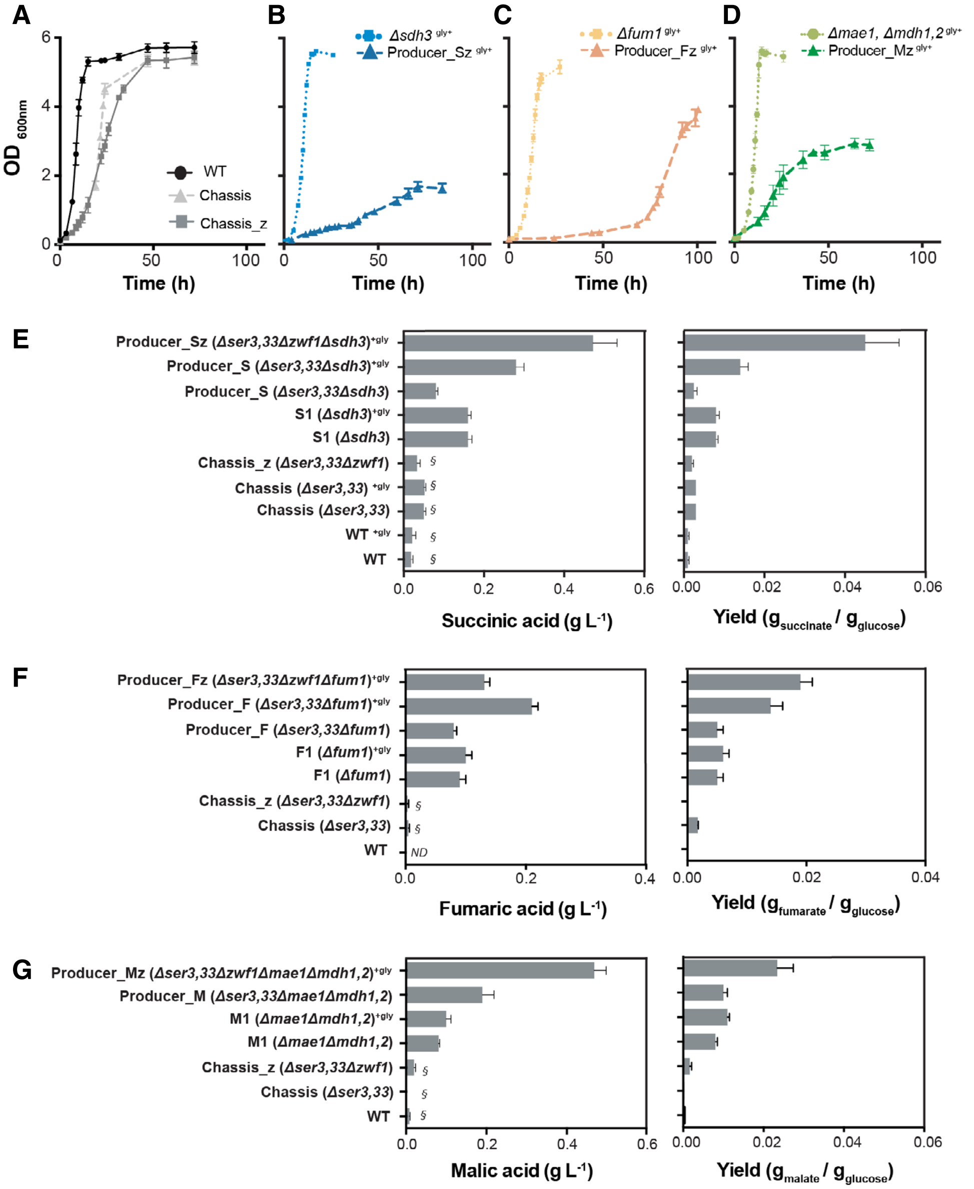

Figure EV2. Growth profile of engineered strains and the impact of the knockouts implemented *in vivo* for the production of target compounds.

A Growth profile of engineered Chassis and Chassis_z compared with WT.

B–D Growth profile of chassis-derived producing strains compared with the strains disrupted solely in the TCA cycle for succinate (B), fumarate (C) and malate (D) production.

E–G Production (left) and yield on glucose (right) of succinate (E), fumarate (F) and malate (G). ND—stands for compounds that were not detected in given conditions, and §—indicates compounds that are measured but were not quantified.

Data information: Cells were cultivated in defined minimal medium with 20 g/l of glucose, with ("+gly") or without glycine supplementation. Average values are shown and error bars represent the standard deviations, $n = 3$ biological replicates.

Source data are available online for this figure.

©2021 The Authors

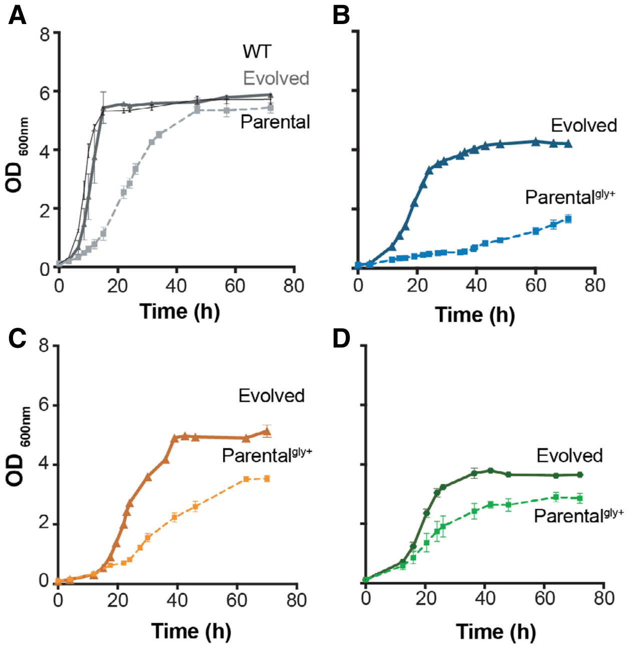

Figure EV3. Growth profile of evolved and parental Chassis_z and chassis-derived producing strains.

A Growth phenotype of Chassis_z strains before (grey squares/dash line) and after evolution (grey triangle/full line), compared with the wild-type (WT, black/full line).

B Growth phenotype of Producer_Sz strain before (blue squares/dash line) and after evolution (blue triangle/full line).

C Growth phenotype of Producer_F strain before (orange squares/dash line) and after evolution (orange triangle/full line).

D Growth phenotype of Producer_Mz strain before (green squares/dash line) and after evolution (green triangle/full line).

Data information: $n = 3$ biological replicates. Average values are shown, and error bars represent the standard deviations. All cultivations were performed in minimal media with 2% glucose, and parental chassis-derived producer strains were supplemented with glycine (depicted with “+gly”).

Source data are available online for this figure.

© 2021 The Authors

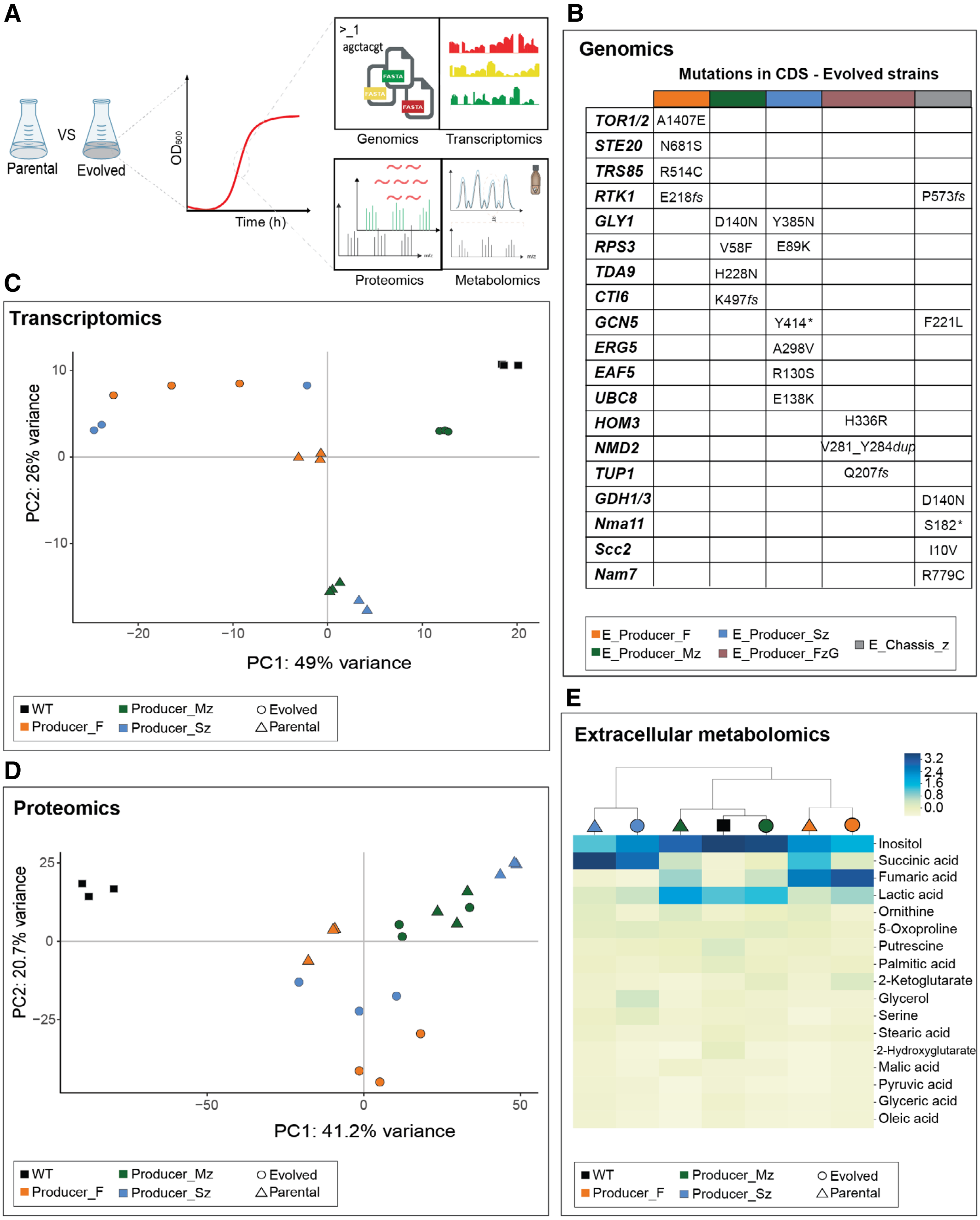

Figure EV4. Multi-omics characterization and data analysis of evolved strains.

A Sample collection approach for multi-omics characterization of evolved strains. Samples of parental and respective selected evolved chassis-derived producing strains were collected for genomics (B), transcriptomics (C), proteomics (D) and extracellular metabolomics (E) characterization ($n = 3$ biological replicates, individual values plotted).

B Whole genome sequencing analysis of SNV and indels identified in evolved strains. Identified mutations are presented by the correspondent amino acid change and position. "fs" represents a frameshift; "*" represents a deletion.

C PCA plot depicting transcriptomics distances between strains. Individual values plotted ($n = 3$ biological replicates, except for Producer_S ($n = 2$ biological replicates)).

D PCA plot depicting proteomics distances between strains.

E Heat map of hierarchal cluster analysis of extracellular metabolomics (GC-MS) analysis of evolved and parental strains. Yellow represents the metabolites with lower AUC, and blue represents the metabolites with higher AUC.

Source data are available online for this figure.

 ©2021 The Authors

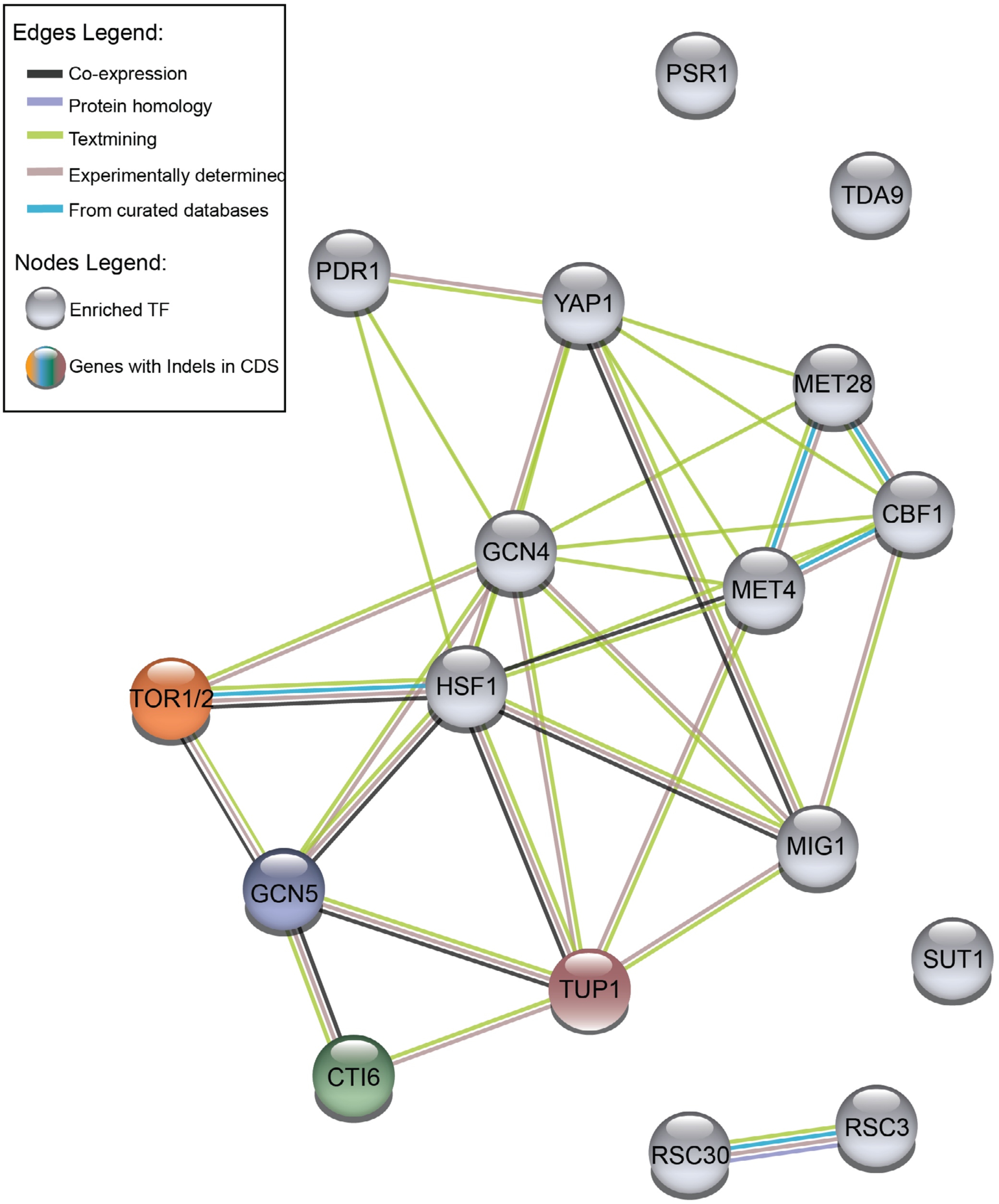

Figure EV5. STRING analysis shows known interaction between enriched TF and the genes with identified mutations in evolved strains.

STRING analysis of interaction between enriched TFBSs in differentially expressed genes in evolved producing strains (grey nodes) and indels mutations identified in the genome of these strains (coloured nodes). E_Producer_F (orange node), E_Producer_Sz (blue node), E_Producer_Mz (green node) and E_Producer_FzG (pink node). Coloured lines connecting nodes represent different evidences of interaction between the respective nodes. Nodes without connected lines are not known to interact with any other node of the query.
